# RNA Synthesis by *in Vitro* Selected Ribozymes for Recreating an RNA World

**DOI:** 10.3390/life5010247

**Published:** 2015-01-20

**Authors:** Lyssa L. Martin, Peter J. Unrau, Ulrich F. Müller

**Affiliations:** 1 Department of Molecular Biology and Biochemistry, Simon Fraser University, Burnaby, BC V5A 1S6, Canada; E-Mail: lyssam@sfu.ca; 2 Department of Chemistry and Biochemistry, University of California, San Diego, La Jolla, CA 92093-0356, USA

**Keywords:** RNA world, ribozyme, origin of life

## Abstract

The RNA world hypothesis states that during an early stage of life, RNA molecules functioned as genome and as the only genome-encoded catalyst. This hypothesis is supported by several lines of evidence, one of which is the *in vitro* selection of catalytic RNAs (ribozymes) in the laboratory for a wide range of reactions that might have been used by RNA world organisms. This review focuses on three types of ribozymes that could have been involved in the synthesis of RNA, the core activity in the self-replication of RNA world organisms. These ribozyme classes catalyze nucleoside synthesis, triphosphorylation, and the polymerization of nucleoside triphosphates. The strengths and weaknesses regarding each ribozyme’s possible function in a self-replicating RNA network are described, together with the obstacles that need to be overcome before an RNA world organism can be generated in the laboratory.

## 1. The RNA World Hypothesis

The RNA World hypothesis [1,2,3,4] was developed in 1968 to solve the following question: How could modern life have originated on the early Earth? The main problem is that life depends on three completely interdependent systems of aperiodic polymers. DNA, which stores genetic information, requires protein catalysis for its replication, while protein catalysts depend on DNA for their genomic information via the transcription and translation of mRNA. Such an entwined, three-polymer system would have been vastly unlikely to have originated by chance during the early evolution of life. This paradox of interdependent polymers was famously solved by postulating the RNA World hypothesis, which states that today’s DNA-RNA-protein life forms evolved from ancestors in which RNA served both as the genome as well as the only genome-encoded catalyst. This hypothesis was based on the postulate that RNA was able to catalyze chemical reactions—which had no experimental support in 1968. The discovery of two catalytic RNAs (a self-splicing RNA in *Tetrahymena thermophila* and RNase P) in 1982 and 1983 [5,6]) placed the RNA World hypothesis on a more solid footing.

Further support for the RNA World hypothesis comes from “molecular fossils” in today’s life forms: (i) Proteins are synthesized in every known organism by the ribosome, a large RNA/protein complex. It is the RNA portion that catalyzes peptide bond formation [7,8], demonstrating that catalytic RNAs must have existed before ribosome-mediated protein synthesis evolved; (ii) Essential metabolic cofactors, such as NAD^+^, FAD, CoA, SAM and ThPP contain functional groups that are either attached to adenosine or strongly resemble the structure of nucleotides. Since there is no functional requirement for a cofactor to be a nucleotide derivative, they are thought to be remnants from an ancestral form of life in which RNA was the dominant macromolecule [9]; (iii) the synthesis of (DNA) deoxynucleotides in today’s organisms proceeds via (RNA) nucleotide intermediates [10]: The 2'-hydroxyl group is removed from nucleoside triphosphates to generate 2'-deoxynucleotide triphosphates, and the C5-methyl group is attached to 2'-deoxyuridine triphosphate to generate 2'-deoxythymidine triphosphate. Further, nearly all extant DNA polymerases require the synthesis of RNA primers by DNA primase to commence strand synthesis in DNA replication. This biochemical evidence further supports the idea that RNA is evolutionarily older than DNA. Though the existence of an early RNA world is well-supported, several central problems remain unsolved, including the prebiotic synthesis of nucleotides and RNA polymers, and the demonstration in the lab of a self-replicating and evolving system based on catalytic RNAs [11].

**Figure 1:**
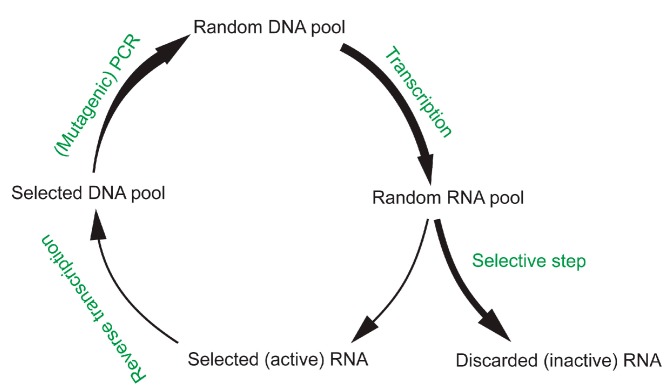
Schematic for the *in vitro* selection of catalytic RNAs. A pool of double-stranded DNA molecules containing random sequence is transcribed into the corresponding RNA pool. Due to the differences in sequence and self-complementarity, the individual sequences of the RNA pool fold into distinct three-dimensional structures. To identify the tiny proportion of these structures that are able to catalyze the reaction with a substrate the RNA pool is incubated with the substrate and the unreacted RNAs are removed in a selection step. The isolated RNAs are then reverse transcribed into DNA molecules, and amplified by PCR to obtain a DNA pool that is now enriched for sequences that are able to catalyze the desired reaction. Because the complexity of the initial DNA pool (usually 10^14^–10^16^) vastly outnumbers the enrichment of active sequences per selection cycle (usually 10–10^4^-fold) it is necessary to conduct multiple cycles before a detectable fraction of the pool shows catalytic activity.

Using the method known as *in vitro* selection [12,13] more than a dozen research groups have demonstrated that catalytic RNAs (ribozymes) have the potential to catalyze the diverse chemical reactions required to sustain a metabolism (for a review, see [14]). Such *in vitro* selections start from large, combinatorial libraries of RNA that are incubated with their substrate/ligand molecules (Figure 1). For the *in vitro* selection of catalytic RNAs [15], one of the substrate molecules usually contains a handle, which is covalently coupled by the ribozyme to itself. This self-tagging allows reacted RNA molecules to be isolated from the library. Subsequently, the isolated RNA is reverse transcribed into DNA, amplified by PCR, and transcribed into an RNA pool that is now enriched for functional sequences. Multiple cycles of this selection and amplification scheme are necessary to obtain libraries from which active sequences can be isolated by analyzing individual clones. Note that some *in vitro* selections employ more complicated schemes for isolating active sequences from the library instead of a simple handle on the substrate (for examples, see [16,17]) or bypass the step of reverse transcription [16]. With this method of *in vitro* selection, ribozymes were developed that could catalyze, among many others, reactions that include RNA ligation, peptide coupling, Diels-Alder bond formation, redox reactions and recently decarboxylation ([15,18,19,20,21]; for a review, see [14]). While the catalytic rate enhancements of ribozymes are usually lower than those of highly evolved protein enzymes, the repertoire of reactions catalyzed by ribozymes appears sufficient to mediate a complex metabolism. This supports the plausibility of an RNA World during early stages of life [14]. This review focuses on a subset of *in vitro* selected ribozymes whose activities could directly mediate the synthesis of RNA from simpler building blocks.

## 2. RNA Synthesis in an RNA World

**Figure 2:**
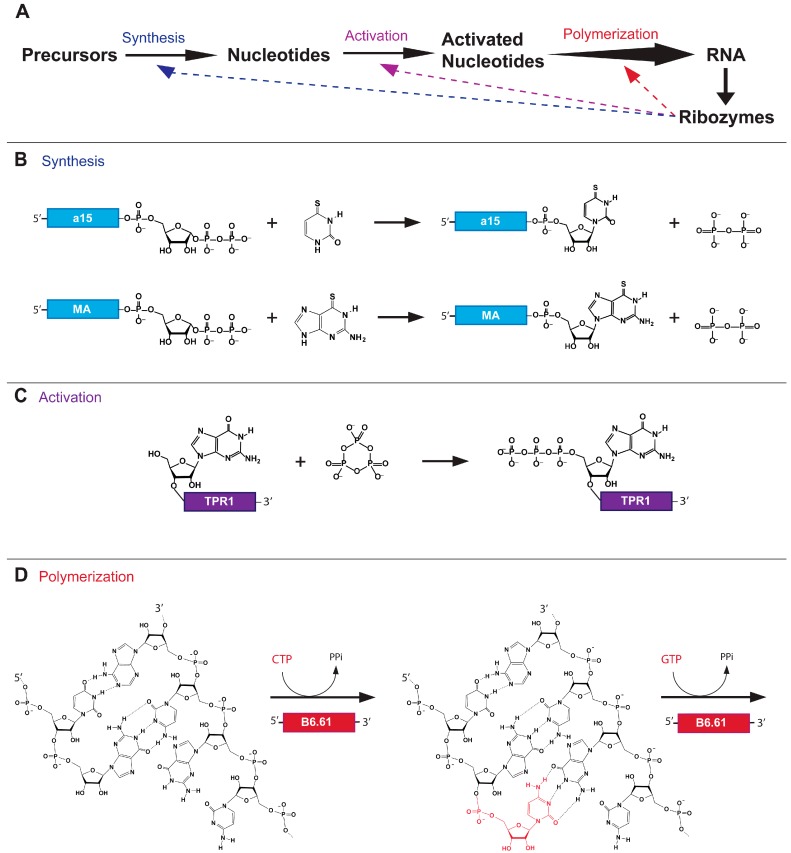
Steps in the synthesis of RNA before and during the RNA World. (**A**) Prior to the emergence of RNA catalysts, abiotic processes led to the generation of RNA polymers (black arrows). After the emergence of RNA catalysts some or all of these reactions were catalyzed by ribozymes (dotted lines); (**B**) Reactions catalyzed by nucleotide synthase ribozymes. The a15 ribozyme promotes the synthesis of ^4S^UMP from free ^4S^Ura and PRPP tethered to its 3'-terminus. Similarly the MA ribozyme catalyses the synthesis of ^6S^GMP from free ^6S^Gua and tethered PRPP; (**C**) The TPR1 ribozyme promotes the triphosphorylation of its 5'-terminus with trimetaphosphate; (**D**) Template dependent RNA polymerization is catalyzed by the B6.61 RNAP ribozyme.

The first catalytic RNA must have arisen in an environment that already executed all steps in a chemical pathway from prebiotically available molecules to RNA polymers—otherwise this catalytic RNA could not have existed (Figure 2A). The chemistry of RNA polymer synthesis before the emergence of RNA World catalysts is poorly understood and has been proposed to occur in several alternative ways. Nucleotide synthesis has been suggested to have proceeded via the glycosidic bond formation between ribose and nucleobases [22,23] or the stepwise assembly of a nucleobase on ribose [24,25]. Many routes of prebiotic nucleotide polymerization have been explored. Some of these routes do not require activation groups on the nucleotides: such as the formation of polymers by the dehydration of nucleoside 5'-monophosphates in lipid environments [26], and the formation of dinucleotides by the reaction of adenosine 2',3'-cyclic phosphate on poly(U) templates [27]. The latter of these studies analyzed the balance between dinucleotide formation and hydrolysis under different conditions, illustrating the thermodynamic challenges of RNA polymerization. In aqueous solution, RNA polymerization is entropically disfavored, making activating groups necessary for the production of long RNA polymers. A range of 5'-phosphate activating groups with variable prebiotic plausibility have been investigated: adenylate [28], cyanide [29], imidazole [30], 2-methyl imidazole [31] and triphosphates [32]. While these forms of chemical activation have provided insight into RNA polymerization, polyphosphates are considered the most prebiotically likely activation groups [33,34]. The polymerization of activated nucleotides into RNA polymers has been explored on the surface of clay minerals [35] or using cations such as Zn^2+^ [30]. It is currently unclear which of these possibilities, if any, preceded the chemical pathways of the RNA World. The first RNA world organisms likely used existing abiotic chemical pathways and improved on their efficiency and/or selectivity by catalyzing rate-limiting reactions. Later evolutionary stages of RNA World organisms may have modified these pathways to use better suited metabolites essential for RNA replication (Figure 2A). With the onset of the RNA World, vesicle-encapsulated aqueous droplets would have served as the center for an RNA metabolism sustained by ribozymes encoded by an RNA genome [36,37], placing constraints on the chemistry and kinetic stability of nucleotide activation groups in aqueous solution.

The takeover of prebiotic chemistry by RNA world ribozyme catalysis increased the rate and specificity of RNA synthesis, thereby allowing faster replication of the RNA world organisms (Figure 2A). To recapitulate an RNA World organism in the lab, three types of ribozymes have been identified by *in vitro* selection methods, and characterized: Ribozymes that synthesize nucleotides from activated ribose and nucleobases [38,39] (Figure 2B), ribozymes that convert RNA 5'-hydroxyl groups to 5'-triphosphates [40] (Figure 2C), and ribozymes that catalyze the template-dependent polymerization of nucleoside 5'-triphosphates to RNA polymers [41,42], (Figure 2D). These three types of ribozymes are the focus of this review. All of these ribozymes use phosphate activation groups to mediate steps in a pathway for RNA synthesis that may have existed in the RNA world.

## 3. Nucleotide Synthesis by RNA Ribozymes

A nucleotide is a fusion of three discrete components: a phosphate group, a ribose sugar, and a nucleobase. These elements combine to give RNA its information encoding and catalytic properties. The preferred conformations of ribose together with the 5'-3' phosphodiester linkages formed by polymerization stabilize purine and pyrimidine nucleobase pairs. The chemistry, size, and geometry of the resulting polymers is fine-tuned for the storage and transmission of genetic information via base pairing [43]. Nucleoside triphosphates, the activated monomers used in biology for RNA polymerization, are also used metabolically, as central sources of cellular free energy. Together with canonical base pairs, abundant alternative purine and pyrimidine hydrogen bonding patterns [44], and sugar-base conformations make possible a broad range of alternative RNA structures which are required for the formation of complex catalytic RNAs [45,46,47]. The discrete elements of a nucleotide beg the question: did the synthesis of nucleotides in an RNA World reflect the three-fold modularity found in modern metabolism?

In modern organisms, 5-phosphoribosyl 1-pyrophosphate (PRPP) is essential for all nucleotide synthesis. PRPP, which is obtained from ribose 5-phosphate (R5P) and ATP, brings together phosphate based energy metabolism with ribose carbohydrate chemistry. PRPP then reacts with nucleobases that are either scavenged or synthesized by nitrogen dependent metabolic pathways to yield a nucleotide product linking R5P and a nucleobase. Both the information containing and catalytic cofactor type nucleotides are synthesized by this strategy. Purine nucleobases such as adenine, guanine, xanthine, hypoxanthine, and 6-thioguanine can be reacted in a single step with PRPP to produce their corresponding nucleotide monophosphates (NMPs). Alternatively, the first step in *de novo* purine nucleotide synthesis aminates the 1-position of ribose 5-phosphate (R5P) using PRPP as substrate [48]. Pyrimidine nucleotides such as UMP can be produced directly by reacting PRPP with uracil, but are primarily produced via the orotate dependent pathway, where orotodine is produced in a single concerted reaction. Subsequent decarboxylation at the 5-position then yields UMP, and can be followed by amination at the 4-position to produce CMP. Likewise pyrimidine nucleobases such as nicotinamide and nicotinate are used to synthesize NAD^+^, an essential redox cofactor. PRPP considerably simplifies nucleotide synthesis in modern metabolism by providing two of the three essential elements of a nucleotide in the form of activated R5P.

Modern biology’s dependence on PRPP, and the advantages of modular RNA nucleotide synthesis with PRPP suggest that this molecule might have played an important role in a potential RNA World. However, promoting PRPP dependent nucleotide synthesis chemistry with RNA presents a potential problem: both PRPP and the nucleobases used to make nucleotides are small relative to the size of the catalytic RNA itself, making it unclear if RNA is up to the task of nucleotide synthesis. Specifically, it is a challenge for RNA to position its functional groups necessary for specific ligand binding and catalysis of a reaction into the tight space of a binding pocket for a small molecule. While riboswitches and RNA aptamers that specifically bind and recognize adenine, guanine, and derivatives are now well-understood [49,50,51] few naturally occurring RNAs are known that chemically manipulate small substrates. Among these few is the GlmS glucosamine-6-phosphate (G6P) ribozyme, which utilizes bound G6P to self-cleave by promoting an acid-base mediated cleavage reaction [52]. The combination of small substrate binding and catalysis may be possible for the GlmS ribozyme because self-cleavage is intrinsic to all RNAs: It only requires a mechanism to deprotonate and position a 2'-hydroxyl in line with its adjacent phosphodiester bond; an event that proceeds at measurable rates at most positions in almost all RNA molecules. Given the limited chemical repertoire of the ribozymes that manipulate small molecules, the isolation of ribozymes in the laboratory provides one of the few tools to study the potential of RNA to perform reactions like nucleotide synthesis that could have played an essential role in an RNA World.

Encouragingly *in vitro* selections for pyrimidine and purine nucleotide synthesis have demonstrated the potential for RNA to promote nucleotide synthesis (Figure 2B). Selections were designed where large pools (~10^15^ distinct sequences) of random sequence RNA were ligated to PRPP via their 3'-termini. These pools were then incubated with either 4-thiouracil [38,53] or 6-thioguanine [39]. Those RNAs able to promote glycosidic bond formation between tethered PRPP and thiolated nucleobase were purified using polyacrylamide gels derivatized with *N*-acryloylaminophenylmercuric acetate [54]. Purine nucleotide synthase pool populations were found to be some 50 to 100 times more efficient than the equivalent pyrimidine nucleotide synthase ribozymes [39]. Consistent with the bulk pool reaction rates, the three most common pyrimidine nucleotide synthase families named A, B and C had apparent efficiencies of 4.3, 1.3, and 0.7 M^−1^·min^−1^, while the two most abundant purine nucleotide synthase families named RA and MA had apparent efficiencies of 230, and 284 M^−1^·min^−1^ respectively. Only the pyrimidine family A and purine family RA had measurable *K*_m_ values with the remaining families having reaction rates directly proportional to nucleobase concentration. This suggested that mechanistically, ribozymes isolated from purely random sequence can invest in either substrate binding or in chemical rate enhancement but have difficulty optimizing both binding and rate acceleration simultaneously. Consistent with this hypothesis reselection and truncation of the pyrimidine nucleotide synthase family A ribozyme improved chemical, apparent efficiency from 4.3 to 150 M^−1^·min^−1^ [55] and it is possible that similar gains could be obtained for purine nucleotide synthase ribozymes should such reselections be attempted in the future.

**Figure 3:**
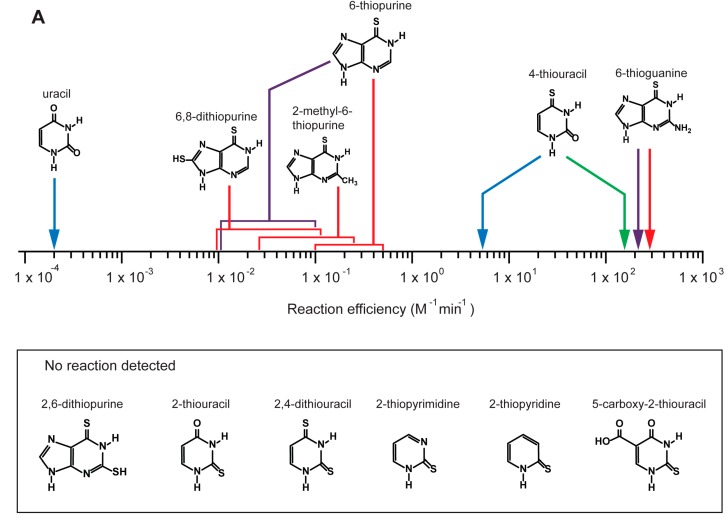
Pyrimidine and purine nucleotide synthase ribozymes show good substrate discrimination. The arrows point to reaction efficiencies for ribozymes with the indicated substrate molecule: red arrows indicate the MA purine nucleotide synthetase ribozyme reaction efficiencies, purple indicates the RA purine nucleotide synthetase ribozyme, blue indicates the Family A pyrimidine nucleotide synthethase ribozyme and green indicates the improved a15 ribozyme derived from the Family A ribozyme by reselection. In the box nucleobases for which no activity was detected are shown.

Whether or not a nucleotide synthase ribozyme had measurable nucleobase binding affinity, all families of nucleotide synthetase ribozymes were extremely sensitive to nucleobase modification and showed surprising parallels to the substrate preferences of highly evolved protein nucleotide synthase enzymes. For example, the family A pyrimidine nucleotide synthetase ribozyme was ~10,000 times slower with uracil than with 4-thiouracil and reactivity with 2-thiocytosine, 2-thiopyrimidine, 2-thiopyridine and 5-carboxy-2-thiouracil could not be detected (Figure 3). In contrast, the MA purine nucleotide synthetase ribozyme was 600 to 3000 times slower when incubated with 6-thiopurine than with 6-thioguanine; while the RA purine nucleotide synthase ribozyme was 5 to 10 times slower still. As these purine nucleotide synthetase ribozymes were much slower with other purine modifications this indicates a lack of substrate discrimination at the 2-position. A similar situation is seen with the protein enzyme HGPTase, which is also unable to discriminate between guanine and hypoxanthine [56]. Similar patterns are seen in guanine binding riboswitches [57]. That two purine nucleotide synthase ribozymes share patterns in common with these naturally selected protein and RNA systems suggests that a single optimal strategy to specifically recognize guanine by hydrogen bonding and stacking interactions exists in nature.

The differences in apparent ribozyme efficiencies strongly suggest that pyrimidine nucleotide synthesis is the more challenging chemistry compared to purine nucleotide synthesis. This is consistent with the disfavored kinetics and thermodynamics of pyrimidine glycosidic bond formation relative to that of purine nucleotide synthesis [39]. Additionally, the large difference in *K*_m_ between pyrimidine and purine nucleotide synthase ribozymes (28 ± 4 mM for family A and, 78 μM for family RA) suggests that 6-thioguanine is considerably easier to bind (presumably via stacking interactions to the purine base) than 4-thiouracil. Consistent with these barriers to pyrimidine glycosidic bond formation, kinetic isotope measurements performed on the family A pyrimidine nucleotide synthase ribozyme point to an unusually dissociative reaction mechanism, where a positively charged oxocarbenium-ion is stabilized presumably by the negatively charged phosphodiester backbone of the ribozyme [58].

Given that ribozymes can easily be found that promote nucleotide synthesis chemistry, how might new small molecule chemistry have evolved in an RNA World? If *in vitro* selected ribozymes are modular in nature, then it might be possible to convert a pyrimidine nucleotide synthetase into a purine nucleotide synthase by varying one subdomain, the substrate recognition domain, and conserving the other, catalytic domain. A high diversity set of RNAs whose sequences where derived from that of the family A pyrimidine nucleotide synthetase was constructed to test this hypothesis explicitly, but found no evidence for functional modularity [39]. Instead the secondary structure of the family, a ribozyme had been disrupted by a series of point mutations able to form completely new RNA folds. This complete change in RNA structure provides additional support for the theory of “neutral networks” where high densities of distinct RNA folds (and hence functionalities) are highly proximal in sequence for RNAs of sufficiently small size space. This allows the efficient evolution of RNA functionalities with domains that are sufficiently small [59]. Later in evolution these domains can be combined to generate multidomain ribozymes such as the ribosome or, as we will see later, convert an RNA ligase into an RNA polymerase [60].

Ribozyme substrate promiscuity may provide an additional mechanism to efficiently evolve new ribozyme functions rapidly. A ribozyme able to react with two alternative substrates can in principle be evolved into two distinct ribozymes, each optimal for its own substrate. Therefore, promiscuity could be exploited to rapidly populate an RNA world with activities that can be related to each other by promiscuity. Such an evolutionary mechanism is particularly interesting if ribozymes able to “metabolize” simpler RNA relevant substrates can, via promiscuity, naturally evolve to utilize substrates that carry a higher free energy potential and are more similar to today’s metabolites than the initial substrates. Experimental support for such a model was found unexpectedly by the selection of a ribozyme able to react tethered R5P to 6-thioguanine via an aldehyde dependent chemistry [61]. Surprisingly, changing the R5P substrate to PRPP allowed efficient nucleotide synthesis chemistry to occur with 6-thioguanine, even though this nucleotide synthesis chemistry had never been selected for. The directionality of this promiscuity was not general in the sense that ribozymes selected for their PRPP dependent nucleotide synthase ability failed to react with R5P. Since R5P and PRPP are fundamental to modern RNA metabolism and are likely to have been important in an RNA world, it is interesting to speculate how many other examples of such promiscuity can be found with other important biomolecules in the future.

## 4. Triphosphorylation Ribozymes

Nucleotides require chemical activation to drive the entropically disfavorable RNA polymerization in aqueous medium. All known life forms use nucleoside 5'-triphosphates as energy currency, therefore the use of nucleoside 5'-triphosphates by RNA World organisms would fit well with their ancestry to today’s life forms. Assuming that nucleosides were synthesized on prebiotic Earth, how might they have been converted to 5'-triphosphates? Long, linear polyphosphates are ubiquitous in microbes and animals, serve multiple biological roles, and are a potential remnant of the RNA world [62]. Polyphosphates are prebiotically plausible activation groups of nucleoside 5'-phosphates partially because polyphosphate-activated nucleotides are kinetically stable in aqueous solution [63]. Cyclic trimetaphosphate (Tmp) is the most active polyphosphorylating reagent of all polyphosphates [64] and it is one of the more abundant polyphosphates that are generated by three prebiotically plausible synthetic routes [65,66,67], most importantly by the synthesis from the meteoritic mineral Schreibersite, or (Fe,Ni)_3_P [68]. Tmp reacts with adenosine at room temperature to produce about 5% 5'-triphosphorylated adenosine at a pH of 12 [32]. The phosphorylation of the 2'- and 3'-hydroxyl groups is much more efficient [69] because these hydroxyl groups have pK_A_ values in the range of 12.3 while the 5'-hydroxyl groups has a pK_A_ around 15 [70,71] but 2'- or 3'-triphosphorylated nucleosides quickly decompose to form 2',3'-cyclic phosphates and pyrophosphate [34]. An additional limitation of the yield in triphosphorylation reactions appears to be the hydrolysis of Tmp at high pH [72]. The biggest problem with the necessity for pH 12 in this nucleoside triphosphorylation reaction is that RNA polymers hydrolyze rapidly at this pH. Therefore, the triphosphorylation of nucleosides in an RNA world organism would require a catalyst that facilitates the reaction near neutral pH.

To obtain ribozymes that catalyze the triphosphorylation of RNA 5'-hydroxyl groups near neutral pH (Figure 2C), an *in vitro* selection from a diverse library (~10^14^) of RNA sequences was performed [40]. The RNA library was challenged to triphosphorylate their 5'-hydroxyl groups using trimetaphosphate. To facilitate this, the RNA pool molecules were prepared with a 5'-hydroxyl group with the help of a cis-cleaving hammerhead ribozyme [73]. After incubation of the RNA pool with trimetaphosphate, pool molecules that affected their own 5'-triphosphorylation were isolated by the use of a ligase ribozyme [74]. The ligase ribozyme linked 5'-triphosphorylated RNAs to the 3'-terminus of a biotinylated RNA oligonucleotide, which allowed the capture of active RNAs by streptavidin. Active clones were isolated after 5 cycles of the selection, and all but two of the 36 clones isolated (16 after 5 cycles, and 20 after 8 cycles of the selection) catalyzed the triphosphorylation reaction. Most of these sequences were unrelated to each other, suggesting that on the order of 100 different triphosphorylation ribozymes were obtained in this selection. Because the effective pool size in the selection was around 10^14^ this suggests that about 1 in 10^12^ random sequences with a length of 150 nucleotides is able to catalyze the triphosphorylation of their 5'-hydroxyl groups. These results showed two important points regarding the RNA world: First, catalytic RNAs are able to use Tmp for the triphosphorylation of RNA 5'-hydroxyl groups, which demonstrated that RNA world organisms could have used Tmp as an energy source. Second, triphosphorylation ribozymes are relatively frequent (~1/10^12^ in our library), apparently about 20-fold more than ribozymes catalyzing RNA ligation [15]. This suggests that triphosphorylation ribozymes were at least as accessible to RNA world organisms as ligase ribozymes, which are also required in an RNA world (see next chapter).

**Figure 4:**
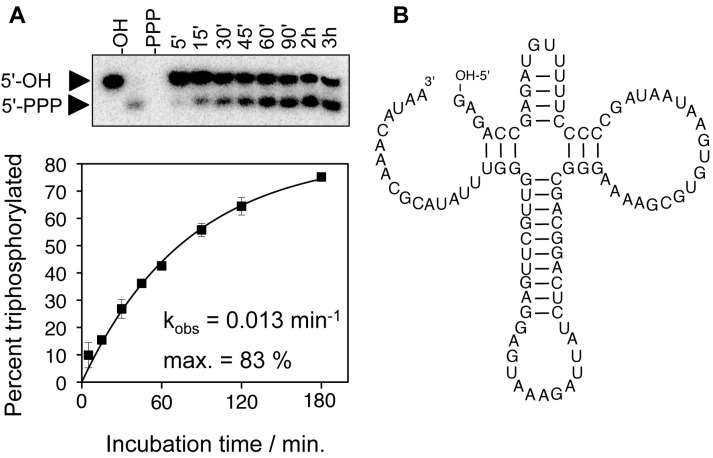
Reaction and secondary structure of a triphosphorylation ribozyme. (**A**) Gel shift assay of the triphosphorylation reaction at different reaction times with Tmp (5 min to 3 h). An 8-nucleotide fragment is cleaved from the 5'-terminus of the ribozyme after incubation with Tmp. The short length of this fragment allows separating the fragments with a 5'-terminal hydroxyl group (5'-OH) and with a 5'-triphosphate (5'-PPP). The percent of the fragment that are triphosphorylated are plotted as a function of the incubation with trimetaphosphate, which allows determining the single-exponential reaction rate; (**B**) Secondary structure of TPR1 resulting from Shape probing and base covariation experiments.

Eight of the selected triphosphorylation ribozymes were analyzed for their reaction kinetics, and had rates between 0.013 min^−1^ and 0.028 min^−1^, under selection conditions (50 mM Tmp, 100 mM total MgCl_2_, pH 8.3) [40]. One of the ribozymes reacted to 83% and was analyzed in more detail. It was truncated from 182 nucleotides to 96 nucleotides while maintaining full activity. This truncated ribozyme was termed TPR1 (triphosphorylation ribozyme 1). Under optimal conditions (100 mM Tmp, 500 mM total MgCl_2_, pH 8.1) the TPR1-catalyzed reaction rate was 0.16 min^−1^, about 10^7^-fold faster than the uncatalyzed reaction. The *K*_m_ for Tmp was 30 mM, leading to an apparent catalytic efficiency of 5.3 M^−1^·min^−1^. A modification at its 5'-terminus allowed the reaction to proceed *in trans*, allowing a 14-nucleotide long RNA oligonucleotide to be triphosphorylated at its 5'-terminus by the ribozyme. This allowed the confirmation of the triphosphorylated product by mass spectroscopy. The dependence of the rate on the Mg^2+^ concentration suggested that each Tmp molecule coordinated one Mg^2+^ ion in a bidentate fashion and a second Mg^2+^ ion with the remaining negatively charged oxygen. The pH dependence of the reaction kinetics showed that a single deprotonation step was rate-limiting for the reaction, presumably the deprotonation of the 5'-hydroxyl group that made the nucleophilic attack on the trimetaphosphate. The secondary structure of the triphosphorylation ribozyme was analyzed using SHAPE analysis [75] and base covariation analysis (Figure 4). The ribozyme appears to be highly compact, with the help of a 4-way helical junction. It is currently unclear how this 4-way helical junction is arranged in three dimensions, and how Tmp is bound by the ribozyme.

**Figure 5:**
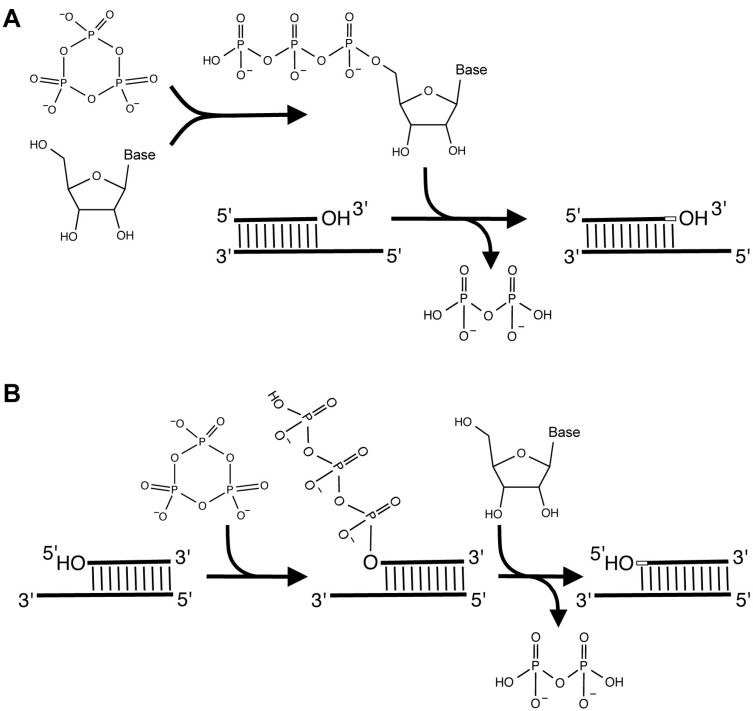
Two alternative routes to RNA polymerization in an RNA World. (**A**) RNA polymerization could have proceeded in a fashion analogous to modern DNA or RNA synthesis, where nucleoside triphosphates are first formed, then react with the primer 3'-hydroxyl groups to elongate the primers in 5'- to 3'-direction; (**B**) Alternatively, RNA World organisms could have relied on RNA polymerization in the 3'- to 5'-direction: here the primer 5'-hydroxyl group is first triphosphorylated, then the activated primer reacts with a nucleoside 3'-hydroxyl group to extend the primer in 3'- to 5'-direction. Note that the monomer in (A) carries three negative charges whereas it is uncharged in (B), facilitating stronger binding of the nucleoside to the elongating primer 5'-terminus.

Six of the isolated triphosphorylation ribozymes were tested for their ability to triphosphorylate free nucleosides, instead of RNA oligomers [40]. None of them showed activity for the triphosphorylation of free nucleosides (^14^C-labeled guanosine). However, the triphosphorylation of free nucleosides may not have been necessary for an RNA world organism: Only if RNA polymerization proceeds in 5'- to 3'-direction is it necessary to triphosphorylate free nucleosides (Figure 5A). If one allows for the idea to polymerize in 3'- to 5'-direction [76,77] then the triphosphorylation of an RNA primer by a ribozyme would prepare the 5'-terminus for the addition of a free nucleoside. Polymerization in 3'- to 5'-direction would then occur by the alternating triphosphorylation of the RNA 5'-terminus and the addition of a nucleoside (Figure 5B). This mechanism has not been observed in today’s life forms; in today’s biology it is probably more beneficial to utilize nucleoside triphosphates and thereby proceed in 5'-3'-direction because nucleoside triphosphates are also used as energy currency to power a large diversity of metabolic reactions. In contrast, the simpler metabolism in the earliest RNA world organisms would have made freely diffusing energy equivalents less beneficial and placed a higher reward on efficient RNA polymerization. In the RNA world, RNA polymerization without nucleoside triphosphates could even have had an advantage: Nucleoside triphosphates carry several negative charges, which cause charge-charge repulsion with the templating RNA strand. In contrast, non-phosphorylated nucleosides do not carry negative charges, mediating stronger binding of the monomer to the growing primer strand [78]. In today’s DNA/RNA/protein life forms the negative charges can be easily shielded by a protein’s positively charged residues, but in an RNA world such shielding would have been more difficult because RNA does not contain positive charges at physiological pH. RNA polymerization in 3'-5'-direction with alternating RNA triphosphorylation and nucleoside addition would have avoided this problem.

In summary, the existence of triphosphorylation ribozymes shows that ribozymes are able to utilize trimetaphosphate as an energy source, with the same chemistry that generates nucleoside triphosphate from nucleosides and trimetaphosphate. Future studies will show how these ribozymes can be integrated with ribozymes that generate RNA polymers.

## 5. RNA Polymerase Ribozymes

The central activity of an RNA World organism is RNA polymerization, to facilitate both self-replication and evolution. Self-replication has also been shown for recombinase or ligase ribozymes [79,80,81] but these systems are limited in their evolutionary potential because they assemble from large RNA fragments of defined sequence. In contrast, polymerase ribozymes generate RNA polymers from monomers such as nucleoside triphosphates, which allows for errors to occur at each position during replication. Therefore, polymerase ribozymes allow the evolutionary exploration of sequence space on the single-nucleotide level and have the potential to invent new activities. This open-ended evolutionary feature is crucial for early life forms to give them the potential to evolve into more complex life forms.

**Figure 6:**
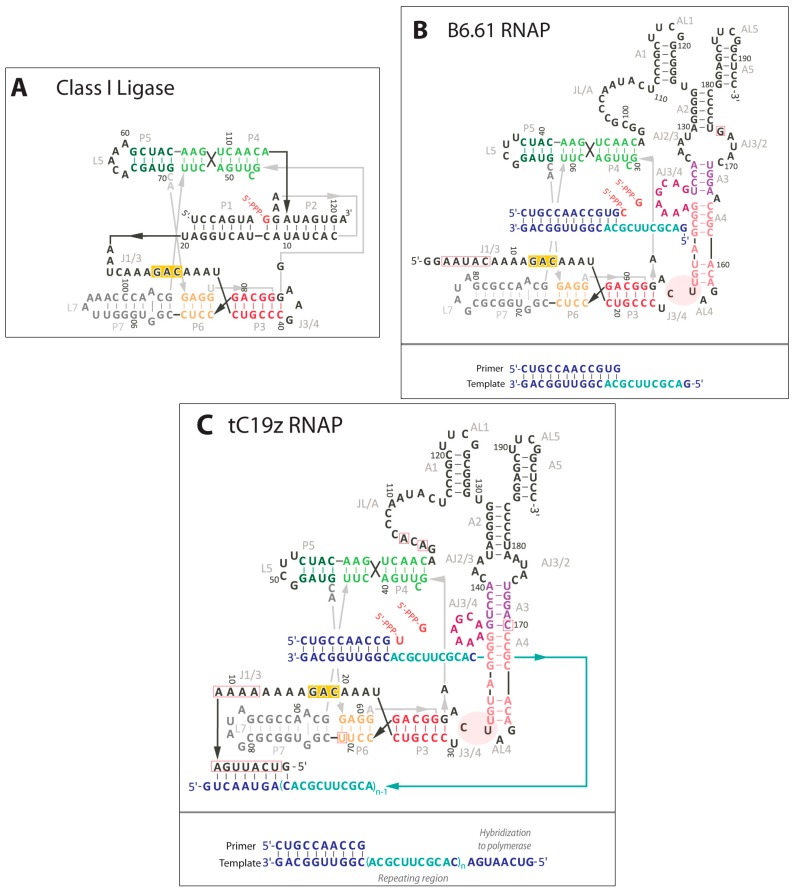
Secondary structure comparison of *in vitro* selected ligase and polymerase ribozymes. (**A**) The Class I ligase secondary structure; (**B**) Proposed secondary structure of the B6.61 RNAP ribozyme in complex with its preferred primer-template (blue) and optimal template sequence shown in cyan. Mutations relative to R18 polymerase are boxed in red; (**C**) Proposed secondary structure of the tC19z RNAP ribozyme in complex with a highly repetitive template sequence (cyan). The 5'-end of the ribozyme was engineered to hybridize to the 3' of the template, promoting longer extension of the primer but also blocking the ribozyme from complete primer extension [17]. Mutations relative to R18 polymerase are boxed in red.

The most successful polymerase ribozyme to date was developed in three stages: First, a ligase ribozyme was developed by *in vitro* selection from a random sequence library containing ~10^15^ different sequences with 220 randomized nucleotides [15]. This ribozyme, termed the “Class I Ligase” (Figure 6A) catalyzes the nucleophilic attack of 3'-hydroxyl groups on RNA 5'-triphosphates, generating 3'-5'-phosphodiester bonds at a rate about 10^7^-fold above that of the uncatalyzed reaction. Second, variants of this ligase ribozyme were designed to extend an RNA primer by six nucleotides, using nucleoside triphosphates [41]. Importantly, the fidelity of these nucleotide additions was 92%, on average. This is much higher than the fidelity estimated from the stability of Watson-Crick pairing (~40%) [41], implying that the ribozyme recognizes to some extent the geometry of a Watson-Crick base pair between the template strand and the incoming nucleoside triphosphate at the catalytic site [82]. Third, an accessory domain was developed for the polymerase ribozyme by *in vitro* selection [42]. To do this, a 76-nucleotide long randomized sequence was appended to the 3'-terminus of the ligase domain. After 18 rounds of *in vitro* selection this library gave rise to the R18 (round 18) polymerase ribozyme, which facilitates the templated primer extension of 14 nucleotides, with an average fidelity of 97%. The R18 ribozyme has been the starting point for reselections which have generated the closely related R18 family: notable members include the B6.61 [16] (Figure 6B) and tC19z RNA polymerase ribozymes [17] (Figure 6C).

The structure of the R18 polymerase ribozyme was characterized by crystallographical and biochemical studies. Two crystal structures of the catalytic core show that the ligase domain of the polymerase ribozyme adopts a tripod-like structure, in which two of the three tripod legs are formed by stem-loops of the catalytic domain and the third leg by the primer/template duplex [82]. The legs of the tripod join at the catalytic center, where the base of the incoming nucleoside triphosphate is stacked onto the primer 3'-terminus and base paired to the first templating nucleotide (Figure 6A). The triphosphate is positioned in a bent conformation at the catalytic site by coordination to several Mg^2+^ ions. The α-phosphate of this nucleoside triphosphate is positioned in a nearly ideal conformation for in-line nucleophilic attack by the primer 3'-hydroxyl group: The distance between the 3'-hydroxyl group and the phosphorus is 3 Å, and the angle between these groups and the oxygen in the leaving group is 176°, close to the ideal 180°. Deprotonation of the 3'-hydroxyl group appears to be mediated by an inner-sphere coordination of Mg^2+^; the negative charge on the pyrophosphate leaving group is stabilized by hydrogen bonds to a ribozyme 2'-hydroxyl group and the exocyclic amino group of a cytosine [82]. The accessory domain of the polymerase ribozyme is draped on top of the tripod-like structure of the catalytic domain [83] (Figure 6B,C). It contains a purine-rich 8-nucleotide bulge that appears to be involved in stabilizing incoming nucleoside triphosphates and is positioned by interactions between the J3/4 stem loop in the ligase core and the AL4 triloop found in the accessory domain. The primer/template duplex is bound by the ribozyme via three well-defined contacts to the 2'-hydroxyl groups of the primer and the template, two on the primer strand (position-2 and -3 relative to the 3'-terminus) and one on the template strand (position-3 relative to the primer 3'-terminus) [84]. Additionally, several positions in the single-stranded portion of the primer/template (position+3, +4, and +5 relative to the primer 3'-terminus) appear to establish weaker, more flexible hydrogen bond(s). The 2'-hydroxyl group at the primer 3'-terminus was important for catalysis, probably by lowering the pK_A_ of the 3'-hydroxyl group [70]. Together, these results generate a structural picture of the polymerase ribozyme; however the precise three-dimensional structure of the accessory domain remains to be determined.

The efficiency of the polymerase ribozyme is limited by its weak affinity to its primer/template (PT) substrate, with an effective dissociation constant in the millimolar range [85]. This causes a release of the primer/template duplex after most additions of a nucleotide to the primer, generating a highly distributive (as opposed to processive) polymerization mechanism. To date five different approaches have been attempted to overcome the limited efficiency of the R18 polymerase family. First, hydrophobic anchors were attached to the ribozyme and the primer/template such that both RNAs were co-localized on micelles [86]. This approach led to a 3- to 20-fold increase of the product yield, depending on the template sequences. Second, incubation of the polymerization reaction below freezing temperature, in eutectic phase, allowed the polymerization of up to 118 nucleotides when done together with the two other approaches (see below): the use of a specific template sequence, and tethering the ribozyme’s favorite primer/template to the ribozyme by base pairing [87]. Eutectic phases benefit the polymerase ribozyme because the reactants are concentrated between the water crystals formed in the freezing process, and the life-time of the ribozyme is elongated at low temperature. Third, it was tested whether single arginine cofactors attached to the ribozyme could improve polymerization efficiency [88]. Although ten different positions on the polymerase ribozyme were tested none of them led to improved polymerization, probably due to the high Mg^2+^ ion concentrations required for polymerase ribozyme activity. Fourth, G/T-rich sequences appended to the ribozyme near the active site caused strong improvements of polymerization efficiency [88]. The templates used in this study were intentionally chosen to be different from the favorite sequence (see below): Without the G/T-rich oligonucleotides the primer was extended only by 3–5 nucleotides; with G/T-rich sequences the polymerization extended to 7–14 nucleotides. The mechanism of these G/T-rich sequences appeared to be base pairing to the template strands with low sequence specificity because G and T can form base pairs to each two other bases. This principle could be exploited further by cofactors that bind RNA without sequence specificity. Fifth, tethering the PT to the ribozyme led to significant increases in product length. Tethering primer templates using a flexible PEG linker via the L5 or L7 loops or to the 5'-terminus of the polymerase resulted in a significant increase in polymerization efficiency, while tethering PT to the accessory domain at a number of sites resulted in only modest enhancements in polymerization [83]. Only one specific 10-nucleotide long template sequence is known to give efficient polymerization (Figure 6B), repeats of which have been demonstrated to yield polymerization of 95 nucleotides (10 repeats, [17]) and 206 nt (19 repeats [87]) when repeats of this sequence were used as template and tethered to the 5'-terminus of the polymerase by direct hybridization (Figure 6C). These reactions demonstrate that there are no steric factors preventing the polymerization of long RNA polymers, consistent with earlier findings with the R18 polymerase [42]. More importantly, this same tethered setup has yielded a functional RNA “transcript” of the hammerhead ribozyme as demonstrated by Holliger’s group [17].

Despite the increases in processivity described above it may be impossible to rid the relatives of the R18 ribozyme from its addiction to its favorite 10-nucleotide template sequence. A solution to this situation may be presented by polymerase ribozymes with different accessory domains, which were *in vitro* selected in similar fashion as the R18 ribozyme [89]. These accessory domains are initial isolates from an *in vitro* selection, but they have not yet been optimized like the R18 ribozyme. Therefore, while the R18 ribozyme family may have reached its fitness peak in the latest evolution experiments, some of the different accessory domains may give rise to polymerase ribozymes that are efficient enough for self-replication. It is also possible that a whole new approach may be needed to break free of the processivity barrier experienced thus far. A recent, elegant study demonstrates a solution to the problem of limited processivity: The cross-replication of two stereoisomeric ribozymes. Because the stereoisomers cannot form a stable Watson-Crick duplex with each other, these ribozymes are able to replicate their respective stereoisomer so that two of these ribozymes can potentially form a self-replicating system [90]. The current version of the system is particularly promising, since it can use nucleoside triphosphates as substrates. At present it requires short oligomers to generate a full-length copy of its enantiomer, but future versions of this approach may form a self-replicating system from a racemic mixture of nucleoside triphosphates.

## 6. Outlook: Outstanding Obstacles to Forging an RNA World

The ribozymes described in the three chapters above promote three types of reactions that might make it possible to generate an RNA World organism in the lab. What remains to be done to obtain such an organism? Three types of challenges remain: First, the three types of ribozymes are not yet efficient enough to sustain a self-replicating system of ribozymes, and second, their substrate specificities are not yet orchestrated to work with each other. For example, the ribozymes promoting nucleotide synthesis from PRPP and pyrimidine or purine nucleobases require tethering of the PRPP substrate to the ribozyme 3'-terminus [38,39]. To work together with the existing triphosphorylation ribozymes [40] the nucleotide synthetase ribozymes would have to be modified (or new ribozymes selected) that react free PRPP with pyrimidine or purine nucleobases. Alternatively, it could be envisioned that the ribozyme-PRPP conjugates act as covalent intermediates that are later released as nucleosides (the 5'-phosphate of the newly created nucleotide could remain as 2',3'-cyclic phosphate on the ribozyme 3'-terminus, perhaps allowing a new ribose to be conjugated). A similar problem is faced by the existing triphosphorylation ribozymes [40]. These ribozymes triphosphorylated the 5'-terminal nucleoside of the ribozyme with trimetaphosphate but were unable to triphosphorylate free nucleosides. If ribozymes generating free nucleoside triphosphates could be generated they would work together with the existing polymerase ribozymes [16,17,42]. Alternatively, it would be sufficient to triphosphorylate the 5'-terminal nucleoside of RNAs if RNA polymerization could proceed in 3'-5'-direction (Figure 5B) instead of the biological 5'-3'-direction (Figure 5A). However, this would require that the triphosphorylation ribozymes act *in trans* on substrate RNAs, and that ligase ribozymes are identified that condensate the RNA 5'-triphosphate with incoming nucleosides. The polymerase ribozyme faces a different problem related to substrate binding: Only one specific template sequence is bound so well that concatemers of this sequence facilitate the polymerization of 206 nucleotides [87]. However, template sequences that are useful for the generation of ribozymes (and therefore self-replication) have so far led to the polymerization of only 24 nucleotides [17]. It may be possible to obtain polymerase ribozymes efficient enough for self-replication by one of the approaches outlined in the last chapter: Different types of tethering between ribozyme and substrate [83,86,88,91]), reactions under different physical conditions [87,91], or the optimization of different accessory domains [89].

A third set of challenges to generating an RNA World organism in the lab stems from general chemical requirements for the components to work together [92]. A potential stumbling block may be encountered when encapsulating ribozymes in a lipid membrane, since most catalytic RNAs require concentrations of Mg^2+^ that lead to the aggregation of lipid vesicles. Luckily, the coordination of Mg^2+^ with citrate was recently shown to protect lipid vesicles while allowing non-enzymatic RNA polymerization to occur [93]. A similar mechanism may be possible to allow ribozyme-catalyzed RNA polymerization in lipid vesicles [94]. Another issue is the relatively low fidelity of template-dependent RNA polymerization, currently in the range of 97%, which may not be sufficient for the stable propagation of genetic information. However, variants of the polymerase ribozyme can have improved fidelity [16], and the effect of polymerization stalling after mismatches may be sufficient to reach the necessary fidelity [95]. Perhaps of greatest significance is the “strand displacement problem”: Namely, all current polymerase ribozymes generate an RNA double strand. Long RNAs are extremely thermostable, and even after heat denaturation the strands are likely to re-form. How can the individual strands be separated, and kept separate, so that they can fold into functional ribozymes? While some ideas have been discussed on these topics [96] no solution yet exists to these difficult problems. These questions show that a series of discoveries have to be made before it will be possible to generate an RNA World organism in the lab.

If we are successful in generating an RNA world organism, what will we learn about the origin of life? How could we use such an “artificial” organism to find constraints on the origin of life? First, it would show us that, indeed, ribozymes are able to generate self-replicating and evolving systems, and thereby support the RNA World hypothesis. Second, RNA World organisms in the lab would make it possible to study them in ways that are currently impossible: The evolution of these simple organisms could be studied by sequencing their genome every few generations and following their mutations. Because these systems would probably consist of less than a dozen catalytic RNAs it would be possible to analyze and understand all mutual molecular interactions—and thereby understand, for the first time, a life-like system on the molecular level. These organisms would also evolve to become more efficient and would show us more efficient ways to construct RNA world organisms, and perhaps simpler and more likely ways how they could have originated.

While the origin of life may have coincided with the origin of the RNA World it should not be forgotten that understanding the origin of life requires understanding the prebiotic chemistry that made the RNA World possible. For example, the prebiotic synthesis of ribose, nucleobases, and nucleotides is debated with very different models [22,25] and it may be a long time until it is possible to generate the necessary compounds for an RNA world in a prebiotically plausible scenario. Some important questions still lack satisfactory answers, such as: how the necessary regiospecificity and stereospecificity could have been achieved, and how the first RNA polymers could have been made in the absence of ribozymes [92]. While the answers may lie many years in the future, the quest to understand how life originated will remain one of the most exciting activities of mankind.
